# Impact of Ramadan Intermittent Fasting on Oxidative Stress Measured by Urinary 15-F_2t_-Isoprostane

**DOI:** 10.1155/2012/802924

**Published:** 2012-10-22

**Authors:** Mo'ez Al-Islam Ezzat Faris, Rand Nidal Hussein, Ref'at Ahmad Al-Kurd, Mohammed Ahmed Al-Fararjeh, Yasser Khalil Bustanji, Mohammad Khalil Mohammad

**Affiliations:** ^1^Department of Clinical Nutrition, College of Applied Medical Sciences, University of Hail, P.O. Box 2440, Hail, Saudi Arabia; ^2^Department of Nutrition, Faculty of Pharmacy and Medical Sciences, Petra University, P.O. Box 961343, Amman, Jordan; ^3^Department of Clinical Pharmacy and Biopharmaceutics, Faculty of Pharmacy, The University of Jordan, P.O. Box 11942, Amman, Jordan; ^4^Department of Medical Laboratory Sciences, Faculty of Allied Health Sciences, The Hashemite University, P.O. Box 591504, Zarqa, Jordan

## Abstract

Fasting and caloric restriction have been associated with reduced incidence of chronic diseases and cancers. These effects have been attributed to reduced oxidative stress. Since Ramadan intermittent fasting (RIF) has been associated with reduced caloric intake, it was hypothesized that RIF would alleviate oxidative stress in healthy volunteers. The study was designed to elucidate the impact of RIF on oxidative stress measured by 15-F_2t_-Isoprostane (15FIP). Fifty healthy subjects (23 men and 27 women) who intended to fast Ramadan were recruited. Urine and serum sampling and anthropometric and dietary assessments were conducted one week before Ramadan (T0), at the end of the third week of Ramadan (T1), and one month after Ramadan (T2). Biochemical measurements included urinary 15FIP, creatinine, and hematological indices. Results revealed that the urinary level of 15FIP measured at T0 was normal, while they showed a significantly (*P* < 0.05) higher level when measured at T1 concomitant with a significant (*P* < 0.05) increase in the body weight and total body fat percent. In conclusion, results suggest that increased body weight is associated with increased lipid peroxidation and oxidative stress, and the impact of RIF on oxidative stress is mediated by the changes in body weight at the end of the month.

## 1. Introduction

Oxidative stress can be defined as an imbalance between the production of reactive oxygen species (ROS) and the antioxidative defense in the body [1]. It is recognized to have a role in a wide variety of human diseases, including cancer, cardiovascular, pulmonary, neurological, renal, and liver diseases, and even the physiological aging process [1]. It was first defined in 1985 as a situation in which increased level of free radicals and ROS generation overwhelms the antioxidant defense system capacity [2]. Reactive oxygen species such as superoxide anion (O_2_^−^) and hydroxyl radicals (OH^•^) are continuously generated as a result of oxygen metabolic processes [2].

Prostaglandin F_2_-like compounds, termed isoprostanes, provided a major advance in assessing oxidative stress and lipid peroxidation [3]. F_2_-Isoprostanes are considered to be sensitive and specific markers for oxidative stress in experimental animals and human diseases [4]. 15-F_2t_-isoprostane (15FIP), one class of isoprostanes, has been recognized as a specific, quantitative, and chemically stable marker of oxidative stress. It can be measured in urine and peripheral blood [5, 6]. Measurement of 15FIP in plasma or urine has been shown to reflect the oxidative stress of the body in patients with a variety of disease conditions [7, 8]. It has also been suggested that the measurement of 15FIP might predict cancer risk and can be used as a biomarker of exposure to relevant carcinogens [8–10]. For instance, increasing urinary 15FIP has been demonstrated to be associated with increased risk of malignant neoplasms such as breast cancer [10].

Intermittent fasting and caloric restriction, or alternate-day feeding, had been shown to increase resistance to toxicity and stress and to prolong maximum life span in experimental animals and humans [11, 12]. A short-term fasting study (7 days) showed a significant reduction in levels of urinary 15FIP among females [13]. Ramadan intermittent fasting (RIF) is considered a unique model of intermittent fasting [14], as food and fluid intake becomes exclusive at nocturnal time without restriction on the type or amount of food intake. Ramadan fasting is associated with alteration in meal frequency, sleep duration, and reduction in physical activity during the day [15]. Many physiological and psychological changes are observed during RIF [14]. Despite the marked changes in food intake habits, some studies showed that RIF has no effect on body weight or body mass index (BMI) [14–17], while other studies showed that RIF is associated with significant weight loss [18–20]. On the other hand, Ramadan fasting showed no significant change in levels of serum malondialdehyde (MDA) as indicators of lipid peroxidation, or protein carbonyl, with a slight significant reduction of lipid peroxidation in erythrocytes [16].

Based on the established relationship between body weight, caloric intake, and oxidative stress [21], we hypothesized that RIF could be associated with reduced oxidative stress expressed by reduced urinary 15FIP. Therefore, the current randomized study was designed to investigate the effect of RIF on oxidative stress in humans using urinary 15FIP as a biomarker of oxidative stress. Because increased oxidative stress was associated with reduced high-density lipoproteins (HDL) and increased low-density lipoproteins (LDL) [22], the study also aimed to explore the impact of RIF on lipid profile and hematological indices.

## 2. Methods and Materials

This study was carried out over three time points: one week before Ramadan (T0), after the end of the third week of Ramadan fasting (on the 22nd day of Ramadan month) (T1), and one month after the end of Ramadan fasting month (30 days from the cessation of Ramadan month) (T2). Before fasting (T0) represents the time point before practicing fasting, during which the body presents in its normal routine conditions for the majority of the year time. However, T1 represents the time point after practicing RIF for three consecutive weeks and is expected to show the impacts of fasting, while T2 is the time point after cessation of RIF by one month and shows to which extent the effect of RIF would extend and become long standing after the end of Ramadan month. The range of fasting hours during the month was about 14-15 hours a day. The female subjects did not fast during their menstrual period as Islamic rules do not permit fasting during the menstrual period. Accordingly, the fasting period in female subjects ranged from 25 to 23 days, while for men it was 30 days.

### 2.1. Subjects

To avoid different confounding socioeconomic and cultural disparities and to maintain sample homogeneity, the enrolled subjects for this cross-sectional study were recruited on voluntary basis from the surrounding community of medium-to-low economic status (Rusaifa city of Zarqa Governorate, Jordan). The inclusion criteria necessitated that subjects were fasting at least 21 days of Ramadan month and were not taking any medication or receiving any medical treatment immediately before or during the study. Exclusion criteria included excluding subjects who have diabetes, hypertension, asthma, or metabolic disorders and who take any supplements including antioxidant vitamins. Female subjects were not pregnant, lactating or receiving contraceptive medications.

Fifty healthy subjects (23 males aged 18–49 years and 27 females aged 18–51 years) were eligible for this study and voluntary completed the study protocol upon a written informed consent. The study protocol was approved by Petra University Committee on Human Research Ethics. A questionnaire was administered, in which medical history and socioeconomic information were obtained through a personal interview with each subject by a well-trained staff member; the study questionnaire was tested for validity, reliability, and reproducibility before conducting the study.

### 2.2. Blood and Urine Sampling

Venous blood and urine samples were collected from the enrolled volunteers during the three time points. Each subject served as his own control by comparing his/her values before Ramadan (T0) with those during (T1) and after Ramadan (T2). Blood and urine sampling was conducted in the private medical laboratory once a time at each time point. Blood and urine sampling were performed early (first 1-2 hours, 10–12 am) during the period of investigation (between 10 am and 4 pm) at each time point. To narrow the time interval for blood and urine sampling and thus to eliminate the effect of day-time on blood and urine variables, volunteers were asked to come early (at 10 am), and blood was first drawn from most of the volunteers according to their arrivals to the laboratory, and then the less time-sensitive assessments (anthropometric and dietary) were subsequently performed during the rest of investigation period. The obtained sera were separated within an hour by centrifuging the blood samples at room temperature for 5 minutes at 4000 rpm (Sigma 183 3-18K lab Centrifuge, Germany). Aliquots of the blood samples were directly analyzed for complete blood count cells using Auto Hematology Analyzer (Mindray 184 BC-3000, China). Urine samples were immediately frozen in special sampling containers at −80°C.

### 2.3. Anthropometric Measurements

During each of the three visits, the anthropometric measurements including body weight and height (Seca, Italy) and body fat percent (GIMA Body Fat Analyzer, Italy) were performed by a well-trained staff member. Body weight was measured at the same time to the nearest 0.1 kg and body height to the nearest 0.01 m. Body mass index (BMI, Kg/m^2^) was calculated, where volunteers with BMI <25 Kg/m^2^ were considered having normal weight and those with BMI ≥25 were considered having overweight [23].

### 2.4. Urine Analysis for 15IFP

At the end of the study, the three frozen samples drawn during the three time points for each subject were thawed at the same time and then used for measurement of 15IFP using the same kit, personnel, and under the same conditions, in order to eliminate sources of errors and to avoid the negative impact of cycled freezing-thawing on urine components. Samples from the same volunteer were measured in the same assay to reduce the effect of interassay variation. All the samples were freeze-thawed once a time. After thawing, samples were centrifuged at 4000 ×g for 4 minutes. After centrifugation two volumes were taken from each sample: 100 *μ*L of urine sample for analysis of 15FIP and 50 *μ*L of urine sample for creatinine determination.

Preliminary experiments showed that collection of samples in this manner had no impact on the urine content of F2-isoprostane [24]. Spot urine samples were used instead of 24 h urine collection, as there is no significant variation in the level of 15FIP between the two methods of collection [2, 25].

Urinary 15FIP levels were analyzed using competitive enzyme-linked immunosorbent assay (ELISA) kits form US Biological (MA, USA) according to the manufacturer's directions and using Biotek Micro plate reader (USA). Samples were analyzed in triplicates. According to the manufacturer, the lower limit of reliable detection is 0.2 ng/mL. A quality control sample consisting of urine pooled from five controls was analyzed with each batch of test samples. The coefficient of variation was <20% (*n* = 50). Urinary creatinine was measured by colorimetric Jaffe reaction by using Spectroscan 80 D UV-Visible Spectrophotometers (UK) [26].

### 2.5. Hematological Measures

Blood samples were analyzed directly for hemoglobin (Hb), hematocrit (Hct), mean cell volume (MCV), mean cell hemoglobin (MCH), and mean cell hemoglobin concentration (MCHC) using Mindray BC-3000 Auto Hematology Analyzer (China). Serum samples were stored at −80°C for the analysis of total cholesterol (TC), high-density lipoprotein (HDL) and low-density lipoprotein (LDL).

### 2.6. Statistical Analyses

Data are presented as means ± standard deviation (SD) unless stated elsewhere. Continuous variables are presented as means ± SD and categorical variables are presented as frequencies and percentages. Unpaired *t *test was used to test oxidative stress biomarker with gender, BMI, and age differences among volunteers and to compare baseline volunteer characteristics based on gender differences. The paired *t* test was used to compare levels of isoprostane, HDL, LDL, Hb, Hct, MCV, MCH, and MCHC. The data were analyzed with SPSS version 16.0.1. (SPSS Inc., Chicago, IL, USA). Statistical significance was defined as *P* < 0.05.

## 3. Results

Fifty healthy volunteers were included in this study, with 23 men (46%) with a mean age of 31.8 ± 9.0 years and 27 women (54%) with a mean age of 36.0 ± 10.0 years. The baseline characteristics of the subjects are presented in Table 1. Males' weights were not significantly different from those of female level. However, body fat percentage and BMI were significantly different between males and females. BMI and body fat percents of females (27.8 ± 5.6 and 31.6 ± 9.4, resp.) were significantly higher than those of males (24.7 ± 3.8 and 14.43 ± 8.5, resp.).

The hematological makers (Hb and Hct) as well as urine creatinine were significantly (*P* < 0.005) higher among males as compared to female subjects. Other hematological markers (MCV, MCH, and MCHC), lipid profile (LDL, HDL, and TC) and urinary oxidative stress marker (15FIP) were not significantly different. 

As shown in Table 2, most of the study population was of normal body weights (BMI <25), while 32% of the volunteers were considered to be overweight with BMI >25. Only 16% (3 females and 5 males) of the volunteers were smokers. No significant differences were found in the levels of 15FIP in the two age groups, between smokers and nonsmokers, or between overweight and normal weight volunteers. The overweight and obese subjects in the overall volunteers had relatively higher, but not significant, levels of 15FIP as compared to normal weight subjects.

Table 3 shows that the overall subjects showed a significant decrease in the mean body weight (71.7 ± 13.7 Kg) during RIF when compared to the baseline (prefasting, T0) levels with a slight reduction in the mean body weights by about 0.8 Kg measured at T1. Body fat percents and BMI values were significantly reduced at T1 as compared to the T0, with a reduction of 2.8% for body fat and 0.3 for BMI. On the other hand, the hematological markers and 15PIF did not differ significantly T1 when compared to the baseline values. However, significant changes were found after RIF in the body fat%, Hb, Hct, MCV, and 15PIF. At T2, the body fat% and 15PIF increased significantly (*P* < 0.05) when compared to T0 values. The levels of Hb, Hct, MCV at T2, however, showed significant reductions as compared to T0.

At T0 urinary levels of 15FIP varied from 0.27 to 7.28 ng/mg creatinine with a mean of 2.33 ± 1.45 ng/mg creatinine. No significant change was observed during T1 compared to baseline values (*P* = 0.411). At T2 urinary 15FIP ranged from 0.2 to 10.84 ng/mg creatinine, with a mean of 3.0 ng/mg creatinine, which was significantly higher (*P* = 0.029) in comparison to baseline levels.

Changes in serum lipid profile are shown in Table 4. The levels of LDL and TC at T0 were similar to T1 and T2. However, HDL levels significantly increased (*P* = 0.006) at T2 as compared to T0, with a 33% increment over the baseline level, and persisted for more than one month after the last test during Ramadan.

Interestingly, volunteers who lost weight during RIF showed a decrease in the level of 15FIP during Ramadan, with a reduction value of about 11% in reference to the baseline value. However, those who gained or maintained body weight during RIF exhibited an increase in urinary 15FIP levels during Ramadan, with an increment with about 19% (Table 5). Nonetheless, none of the changed value was significantly different when compared to the prefasting values.

The majority of subjects (66%) who maintained their body weights showed a significant increase (*P* = 0.026) in their urinary 15FIP levels at T2 as compared to T1, while those who lost or gained body weights did not exhibit significant changes in their 15FIP levels (Table 6).

## 4. Discussion

This study investigated, for the first time, the impact of RIF on the oxidative stress expressed by urinary 15FIP, which is one of the most sensitive and specific biomarkers of lipid peroxidation. This study is of a great interest to understand whether lifestyle behaviors during Ramadan that include altered feeding and sleeping pattern could impact the oxidative stress, which has a role in a variety of chronic diseases.

The major finding of this study is the insignificant change in the level of 15FIP during RIF, with a significant increase after Ramadan, and that the changes in oxidative stress are accompanied with changes in body weight. Also, practicing RIF does not necessarily reduce oxidative stress unless accompanied with weight reduction at the end of the fasting month. 

In the baseline characteristics, the relatively low hematological markers of females are expected due to menstrual blood loss, while the higher urinary creatinine in males is expected due to their higher protein mass and protein turnover rates.

In agreement with previous studies [18–20, 27, 28], our study indicated a significant reduction in body weight during Ramadan. This weight loss could be attributed to the decrease in fat mass% [27] and/or the decrease in fluid intake and the partial dehydration rather than a decrease in energy intake since weight loss was relatively small and retained to baseline values after Ramadan [20].

Moreover, a significant decrease in body fat percent during Ramadan was observed. This could be explained by the regulatory mechanisms that the body activates during fasting such as insulin hyposecretion and increased glucagon. These mechanisms favor a predominant lipolytic state [15], with a higher tendency to utilize fat rather than glucose as a source of energy [19], and hence a higher fat oxidation. The increased fat oxidation during Ramadan was also reported previously [15, 19, 28].

On the other hand, after RIF no effect on body weight or BMI was observed, in contrast to a significant increase in fat mass%. This could be the result of increased fat and carbohydrate intake. Our findings are in agreement with the several previous studies [14, 17, 29–31], which reported a high total energy intake with a constant body weight after Ramadan. BMI and body fat percents of females were significantly higher than those of males in spite the fact that female weights were lower than those of males, a matter that is related to the shorter heights of female in comparison with those of males for BMI, and the genetic makeup of the females for body fat compartment. 

Short-term changes in food supply during Ramadan fasting affect the body weight in a changeable manner, resulting in weight cycling. Short-term changes in caloric intake had been reported to induce fat metabolism by increasing fat oxidation and storing cycle and may be associated with ketogenesis, depending on the type of diet taken [32]. However, this dependence of body fat stores may help the body to reduce body fat content, and thus alleviating the risk of developing cardiovascular diseases.

Studies in the literature have been conflicting regarding the effect of Ramadan on hematological indices. In this study, there was no effect on hematological parameters during Ramadan, which is consistent with those reported by Dewanti et al. [28] and Al Hourani et al. [33]. However, Hb, Hct, and MCV were significantly decreased after Ramadan. This reduction could be explained under the light of the reported decreased intake of dietary energy and accompanied blood-forming nutrients during the month of Ramadan, such as folate [34].

As presented in this study, RIF showed a beneficial effect on lipid profile by significantly increasing HDL level. This effect had been reported by Ibrhim et al. [16] and Lamri-Senhadji et al. [17]. In the current study, HDL elevation persisted for one month after Ramadan cessation, which is consistent with the findings of Adlouni et al. [35] and Mansi [19]. Despite the high-fat intake during Ramadan, this favorable effect on lipid profile was noticed by Mansi [19], who found a positive association between HDL level and fat intake. The importance of the increment in HDL level after RIF arises from the evidence that reduced HDL particles are associated with increased oxidative stress, especially for the metabolic syndrome patients [22]. Thus, this elevation in HDL is postulated to pose a protective effect against oxidative stress. However, results of the current study contradict with those reported in Tunisia, which indicated for increased BMI and reduced HDL/LDL ratio in Muslim subjects [36].

Concerning the biomarker of oxidative stress, baseline levels of urinary 15FIP in our volunteers were higher than the published normal ranges. This could be due to the different methodology, as our measurements were made with enzyme immunoassay, while the published ranges were obtained using mass spectrometry (MS). It is also important to note that the high variation in the level of urinary 15FIP among the study volunteers was also reported by other published studies [37], which indicate the heterogeneity in oxidative stress in human populations. 

Greater oxidative stress in men has been reported by many studies [2, 38]. However, an unexpected finding of our study was the higher urinary levels of 15FIP in females compared to males, which is expected to be caused by the lower levels of urinary creatinine in women compared with men. This gender difference could also be referred to the differences in adiposity and fat mass% which is significantly greater in females. Our results are consistent with those of several authors [37, 39, 40], which demonstrated significantly higher plasma MDA and 15FIP in females compared to males. No significant change in levels of 15FIP in different age groups was noticed. This has been also reported by several investigators [37, 39, 41]. 

Although not significantly correlated, our study demonstrated a positive association between levels of 15FIP and BMI, which comes in agreement with the findings of several researchers [24, 39, 42–45] in which those with higher BMI had higher 15FIP levels at baseline. However, the findings of our study did not support the hypothesis, because practicing RIF did not result in reduced oxidative stress at the end of Ramadan. The finding of this study is the insignificant change in the level of 15FIP during RIF, with a significant increase after Ramadan. This effect is seen when looking at the results as a whole. It has been noticed that volunteers who lost weight also had reduced levels of urinary 15FIP compared to baseline, while volunteers who gained or maintained stable weight had higher levels of 15FIP. The association between the level of 15FIP and weight has been reported elsewhere by Davì et al. [46], who showed that obese subjects had higher levels of urinary F2-isoprostane 8-iso-prostaglandin F2*α* (8-iso-PGF2*α*) than nonobese ones. They also observed a statistically significant reduction of urinary 8-iso-PGF2*α* in obese subjects who successfully achieved weight loss.

In order to explain this association, urinary 15FIP could be viewed as a reflection of compensatory metabolic changes that act to oppose further weight changes [47]. This compensatory mechanism is known as the metabolic adaptation theory [37], which states that weight gain is compensated by an adaptive increase in energy expenditure, while weight loss is compensated by a decrease in energy expenditure. 

Fat oxidation plays an essential role in the change of body weight. For example, the physiological response to weight gain is an increase in fat oxidation, while weight loss is associated with a decrease in fat oxidation level [37, 47]. On the other hand, subjects with reduced rate of fat oxidation usually gain weight [48], while subjects with a greater ability to oxidize fat lose more weight [49]. This interindividual variability in metabolic responses with different abilities to oxidize fat among individuals has been used to explain the findings in over- and underfeeding experiments, in which individuals respond differently—in terms of weight gain or loss—to standardized dietary regimen [47]. Schutz et al. [50] showed that an increase in fat intake has no short-term enhancing effect on fat oxidation and that an increase in body fat mass is first required to activate the adaptive increase in fat oxidation. This study also demonstrated that equilibrium between fat oxidation and fat intake is required before a new plateau of body weight is achieved. 

Based on these studies and the metabolic adaptation theory, it can be concluded that the decrease in levels of 15FIP during RIF among individuals was due to the significant decrease in body weight and fat mass%. However, after Ramadan, individuals maintained a stable weight despite the significant increase in fat mass%. This was accomplished by activation of the adaptive increase in fat oxidation with corresponding significant increase in 15FIP in order to oppose weight gain.

Although previous studies did not correlate the change in weight with isoprostane level, reviewing their results indicates that they agree with the adaptation theory. For example the studies that showed an increase in level of oxidative stress during or after Ramadan also showed increased or stable weight [15, 16], while studies which showed a decrease in oxidative stress showed a decrease in body weight [13, 51].

The main limitations of the study include the small sample size, which implies that the effect of RIF on 15FIP might be confined to the selected participants. The lack of a control nonfasting group deprived the study from the ability to compare the effect of fasting to nonfasting, and thus making it difficult to attribute the changes in oxidative stress to the absolute effect of fasting. In these studies, the control group of nonfasting subjects is difficult to be obtained, because Ramadan fasting is a religious worship; therefore nonfasting people are very scarce and could not be found due to social, psychosocial and religious limitations. In such cases, the baseline values are considered as reference control values for which different changes are compared. Further, the samples before and after Ramadan were nonfasting which limit the ability to compare pre- and postlipid profiles with lipid profile during Ramadan. Another limitation of this study was the use of ELISA rather than GC/MS, the golden standard for isoprostane analysis, due to limitations in time and cost. Furthermore, this study represents a healthy population and it is not representative for diabetic, hypertensive, or dyslipidemic patients.

## 5. Conclusion

In conclusion, this study indicated that Ramadan fasting activates metabolic changes such as increasing fat oxidation, in order for the body to adapt to the alteration in feeding pattern during the month of Ramadan, and to maintain the body composition within the physiological range. The main findings of this study are that the body weight and BMI remained unchanged after Ramadan, with the significant increase in fat mass%, which may be strongly related to the marked variation in qualitative food intake. These changes in eating patterns during Ramadan may be responsible also for the decrease in LDL and the increase in HDL levels and may contribute to lowering the risks of coronary heart disease. Finally 15FIP was found to be positively associated with obesity and to increase significantly after Ramadan which was parallel to the increase in fat mass%.

## Figures and Tables

**Table 1:** Baseline characteristics of the study subjects.

	All (*n* = 50)	Female (*n* = 27)	Male (*n* = 23)	*P* value^a∗^
Age	34.2 ± 9.9	36.0 ± 10	31.8 ± 9.0	0.135
Weight (Kg)	72.5 ± 14.0	69.9 ± 15.2	75.4 ± 12.4	0.178
BMI (Kg/m^2^)	26.4 ± 5.0	27.8 ± 5.6	24.7 ± 3.8	0.029*
Fat mass (%)	24.3 ±12.0	31.6 ± 9.4	14.43 ± 8.5	0.002*
Hb (g/dL)	13.4 ±1.9	12.0 ± 1.5	14.8 ± 1.0	0.000*
Hct (%)	41.7 ± 5.8	37.5 ± 4.3	46.5 ± 2.8	0.000*
MCV (fl)	85.6 ± 8.0	83.5 ± 7.7	88.0 ± 8.0	0.056
MCH (pg)	27.3 ± 2.9	26.5 ± 2.8	28.0 ± 3.0	0.058
MCHC (g/dL)	32.0 ± 0.8	32.0 ± 0.7	32.0 ± 0.92	0.956
LDL (mg/dL)	135.5 ± 56	118.0 ± 55	148.0 ± 55.0	0.235
HDL (mg/dL)	41.2 ± 10.4	42.3 ± 12.5	41.9 ± 7.35	0.930
TC (mg/dL)	209.0 ± 65.7	192.8 ± 59.1	233.4 ± 70.3	0.254
15FIP (ng/mg creatinine)	2.3 ± 1.4	2.5 ± 1.9	2.1 ± 1.1	0.325
Creatinine/urine (mg/dL)	174.0 ± 83.0	138.4 ± 52.0	217.0 ± 93.0	0.024*

Data are expressed as means ± SD.

^a^Independent sample *t*-test. *Significant difference between males and females at* P* < 0.05, *n* = 50.

15FIP: 15-F_2t_-isoprostane; BMI: body mass index; Hb: hemoglobin; Hct: hematocrit; HDL: high-density lipoprotein; LDL: low-density lipoprotein; MCH: mean cell hemoglobin; MCHC: mean cell hemoglobin concentration; MCV: mean cell volume; TC: total cholesterol.

**Table 2:** Urinary 15FIP (ng/mg creatinine) according to subjects age, body weight and smoking status.

	*n* (%)	All	*P* value^a^	Male	Female	*P* value^a∗^
Age						
<30	32 (64%)	2.35 ± 1.44	*P* = 0.97	2.33 ± 1.4	2.36 ± 1.58	0.977
>30	18 (36%)	2.37 ± 1.5		2.12 ± 0.9	2.5 ± 1.8	0.475
BMI						
<25	34 (68%)	2.15 ± 1.12	*P* = 0.58	2.12 ± 1.18	2.18 ± 1.1	0.902
>25	16 (32%)	2.36 ± 1.58		2.09 ± 1.05	2.53 ± 1.86	0.492
Smoking status						
Smoker	8 (16%)	2.15 ± 1.08	*P* = 0.678			
Non-Smoker	42 (84%)	2.39 ± 1.5				

Data are expressed as means ± SD.

^a^By paired *t*-test. *Significant difference at* P* < 0.05.

15FIP: 15-F_2t_-Isoprostane; BMI: body mass index.

**Table 3:** Values of anthropometric, hematological and 15FIP before, during, and after RIF.

Parameter	Before RIF (T0)	During RIF (T1)	After RIF (T2)
Weight (Kg)	72.5 ± 14.0	71.7 ± 13.7^a∗^	72.6 ± 14.0
Fat mass (%)	24.3 ± 12.0	21.5 ± 11.5*	30.3 ± 10.7*
BMI (Kg/m^2^)	26.4 ± 5.0	26.1 ± 5.0*	26.4 ± 5.0
Hb (g/dL)	13.4 ± 1.9	13.2 ± 2.0	12.9 ± 1.9*
Hct (%)	41.7 ± 5.8	41.2 ± 5.7	40.0 ± 6.0*
MCV (fl)	85.6 ± 8.0	85.4 ± 8.0	84.6 ± 8.0*
MCH (pg)	27.3 ± 2.9	27.5 ± 3.0	28.60 ± 9.4
MCHC (g/dL)	32.0 ± 0.79	32.2 ± 0.9	32.20 ± 2.5
15FIP (ng/mg creatinine)	2.33 ± 1.45	2.6 ± 1.55	3.0 ± 2.20*

Data are expressed as means ± SD.

^a^By paired *t* test. *Significantly different from baseline (T0) at *P* < 0.05, *n* = 50.

15FIP: 15-F_2t_-isoprostane; BMI: body mass index; Hb: hemoglobin; Hct: hematocrit; MCH: mean cell hemoglobin; MCHC: mean cell hemoglobin concentration; MCV: mean cell volume; RIF: Ramadan intermittent fasting.

**Table 4:** Variation in lipid profile before and after RIF.

	T0 (*n* = 25)	T2 (*n* = 25)	*P* value^a∗^
TC (mg/dL)	209.0 ± 66.0	211.0 ± 51.0	0.759
HDL (mg/dL)	41.4 ± 10.0	55.0 ± 18.0	0.006*
LDL (mg/dL)	135.5 ± 56.0	123.0 ± 47.0	0.227

Data are expressed as mean ± S.D.

^a^By paired *t*-test. *Significant difference at* P* < 0.05.

HDL: high-density lipoprotein; LDL: low-density lipoprotein; RIF: Ramadan intermittent fasting; TC: total cholesterol.

**Table 5:** Changes in 15FIP according to weight status before and during RIF.

	*n* (%)	15FIP (T0)	15FIP (T1)	% change in 15FIP	*P* value^a∗^
Decreased weight	16 (32%)	2.62 ± 1.7	2.33 ± 1.4	−11%	0.818
Stable weight	34 (68%)	2.19 ± 1.17	2.60 ± 1.7	18.7%	0.37

Data are expressed as means ± SD.

^a^By paired *t*-test. *Significant difference at* P* < 0.05.

15FIP: 15-F_2t_-Isoprostane; RIF: Ramadan intermittent fasting.

**Table 6:** Changes in 15FIP according to weight status before and after RIF.

	*n* (%)	15FIP (T0)	15FIP (T2)	% change in 15FIP	*P* value^a∗^
Decreased weight	7 (14%)	2.10 ± 1.9	3.1 ± 0.64	32%	0.227
Stable weight	33 (66%)	2.3 ± 1.1	3.57 ± 2.2	36%	0.026*
Increased weight	10 (20%)	3.1 ± 1.4	5.33 ± 1.4	42%	0.103

Data are expressed as means ± SD.

^a^By paired *t*-test. *Significant difference at* P* < 0.05.

15FIP: 15-F_2t_-Isoprostane; RIF: Ramadan intermittent fasting.

## Abbreviations

15FIP: 15-F_2t_-Isoprostane
BMI: Body mass index
CR: Caloric restriction
GM/MS: Gas chromatography/mass spectrometry
Hb: Hemoglobin
Hct: Hematocrit
HDL: High-density lipoprotein
LDL: Low-density lipoprotein
MCH: Mean cell hemoglobin
MCHC: Mean cell hemoglobin concentration
MCV: Mean cell volume
ROS: Reactive oxygen species
RIF: Ramadan intermittent fasting
TC: Total cholesterol.
